# FICD activity and AMPylation remodelling modulate human neurogenesis

**DOI:** 10.1038/s41467-019-14235-6

**Published:** 2020-01-24

**Authors:** Pavel Kielkowski, Isabel Y. Buchsbaum, Volker C. Kirsch, Nina C. Bach, Micha Drukker, Silvia Cappello, Stephan A. Sieber

**Affiliations:** 1 Department of Chemistry, Technical University of Munich, Garching, Germany; 2 Max Planck Institute of Psychiatry, Munich, Germany; 3 Graduate School of Systemic Neurosciences, Ludwig-Maximilians-University Munich, Planegg, Germany; 4 Helmholtz Center, Munich, Germany

**Keywords:** Proteomics, Post-translational modifications, Neuronal development

## Abstract

Posttranslational modification (PTM) of proteins represents an important cellular mechanism for controlling diverse functions such as signalling, localisation or protein–protein interactions. AMPylation (also termed adenylylation) has recently been discovered as a prevalent PTM for regulating protein activity. In human cells AMPylation has been exclusively studied with the FICD protein. Here we investigate the role of AMPylation in human neurogenesis by introducing a cell-permeable propargyl adenosine pronucleotide probe to infiltrate cellular AMPylation pathways and report distinct modifications in intact cancer cell lines, human-derived stem cells, neural progenitor cells (NPCs), neurons and cerebral organoids (COs) via LC–MS/MS as well as imaging methods. A total of 162 AMP modified proteins were identified. FICD-dependent AMPylation remodelling accelerates differentiation of neural progenitor cells into mature neurons in COs, demonstrating a so far unknown trigger of human neurogenesis.

## Abstract (web summary)

Protein AMPylation is a post-translational modification whose implications in cellular physiology are not fully understood. Here the authors develop a cell-permeable AMPylation probe and use it to identify new AMP modified proteins and investigate the role of FICD in neuronal differentiation using cerebral organoids.

## Introduction

Introduction of protein PTMs is a tightly controlled and almost ubiquitous process that often modulates critical protein function^1-6^. PTMs such as tyrosination, acetylation and neddylation are known to play a crucial role in the development of the nervous system and in particular of neurons by broadening the diversity of the tubulin and microtubule proteoforms^7,8^.

**Fig. 1:**
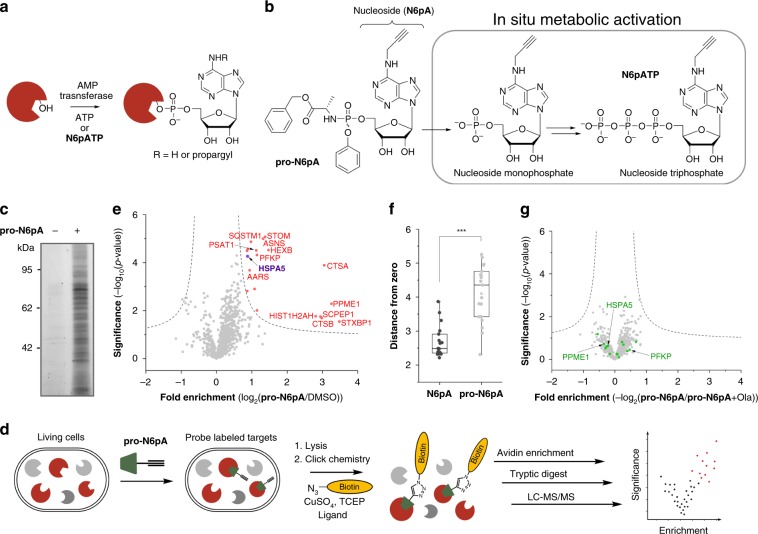
Pronucleotide probe reveals AMPylation of diverse proteins in HeLa cells. **a** AMPylation on Ser, Thr or Tyr. **b** Scheme of the pronucleotide probe **pro-N6pA** and parent adenosine derivative (**N6pA**) and its in situ activation. **c** SDS–PAGE with in-gel fluorescence scanning showing in situ HeLa cell labelling by **pro-N6pA** compared to control (DMSO). **d** Schematic representation of the chemical-proteomic approach used for in situ identification of AMPylated proteins. **e** Volcano plot of fold-enrichment in HeLa cells by **pro-N6pA** labelling compared to DMSO versus significance upon two-sample *t*-test (FDR 0.05, s0 0.3; *n* = 12). **f** Box plot representing comparison of labelling efficiency of pronucleotide **pro-N6pA** (light grey, *n* = 12) and parent nucleoside **N6pA** (black, *n* = 11); Squares represent the mean of the distances to zero for enriched proteins, lines represent the median of the distances to zero and whiskers stand for min and max values. Statistical significance was calculated with two-tailed Student’s *t*-test; ***P < 0.001. **g** Volcano plot of fold-enrichment by **pro-N6pA** labelling compared to probe and Ola treated HeLa cells versus significance upon two-sample *t*-test (FDR 0.05, s0 0.3; *n* = 8). Green circles represent proteins identified as AMPylated in HeLa cells.

AMPylation was first discovered in *Escherichia coli* as regulator of glutamine synthetase activity^9^. Later, it was found that bacterial effectors from *Vibrio parahaemolyticus* and *Histophilus somni* AMPylate Rho guanosine triphosphatases (GTPases) in human host cells^10,11^. These bacterial effectors contain highly conserved Fic (filamentation induced by cAMP) domains, which catalyse the transfer of AMP onto a serine, threonine or tyrosine residue of a substrate protein (Fig. 1a). Approximately 3000 members of this family are known to contain the conserved HXFX(D/E)GNGRXXR sequence motif throughout all domains of life^12^. Despite their abundance in bacteria, only one human protein AMPylator containing the signature Fic domain, termed FICD (also known as Huntingtin yeast partner E, HYPE), has been discovered^12^. Structural and biochemical studies with FICD have revealed that its activity is tightly regulated and controlled by an autoinhibitory loop. Mutation of E234 to glycine overrides autoinhibition and results in a constitutively activated enzyme^12^; the mutant form H363 to alanine is catalytically inactive^4^. One known substrate of FICD is HSPA5, which is a chaperone located in the endoplasmic reticulum (ER) and master regulator of the unfolded protein response (UPR)^3–6^. Recent data show that FICD regulates the ATPase activity of HSPA5 and its interactions with unfolded proteins, but the exact function is not yet clear^13^. However, it was found that the HSPA5 AMPylation associates with changes in neuronal fitness in *drosophila*^3,14–16^.

Just recently, the highly conserved pseudokinase selenoprotein-O (SelO) was found to possess AMP transferase activity in eukaryotic cells^17^. Pseudokinases account for about 10% of the human kinome, but lack the characteristic active site residues and hence their function is largely unknown. However, their putative AMPylation activity is pointing to a possibly larger number of AMPylated proteins in human cells.

Lately, *N*^6^-propargyl adenosine-5′-*O*-triphosphate (N^6^pATP)-derived probes have been applied to profile substrates of AMPylation in cell lysate^18–20^. Nevertheless, the most pressing, unaddressed challenge in discovering the function of AMPylation is the global analysis of AMPylated substrates under physiological conditions inside living cells. Particularly, ATP-derived probes suffer from restricted uptake of the charged nucleotides as well as competition with high endogenous ATP levels. Thus, new concepts are urgently needed to unravel the function of AMPylation in eukaryotic cells.

Here we present a chemical-proteomic approach for identification of protein AMPylation in living cells using a pronucleotide probe and uncover FICD-dependent acceleration of neuronal differentiation in cerebral organoids (COs).

## Results

### Adenosine pronucleotide probe reports on protein AMPylation

To approach the challenge of identifying AMPylated proteins in situ, we selected a phosphoramidate pronucleotide strategy (Fig. 1a, b)^21^. This delivery method not only enhances the probes’ cell membrane permeability but also bypasses the first phosphorylation of the nucleoside analogue by kinases. Based on these considerations, we designed and synthesised a *N*^6^-propargyl adenosine phosphoramidate pronucleotide (**pro-N6pA**, Supplementary Fig. 1). We initiated our investigations with metabolomics experiments to determine **pro-N6pA** in situ metabolic activation to the corresponding **N6pATP**. A maximum concentration is reached 8 h after **pro-N6pA** addition and it is maintained for at least 24 h (Supplementary Fig. 2). For the subsequent analysis of AMPylated proteins, we treated living (intact) HeLa cells with **pro-N6pA** (100 µM in DMSO) or dimethylsulfoxide (DMSO). Subsequent click-chemistry to a rhodamine-biotin-azide tag, enrichment on avidin beads and SDS-PAGE analysis via in-gel fluorescence detection revealed several distinct protein bands in the soluble fraction (Fig. 1c). Next, we performed quantitative proteome profiling in HeLa cells^22^. Enriched proteins were trypsin digested and resulting peptides were either isotopically marked by dimethyl labelling (DiMe) prior to LC-MS/MS measurement or analysed directly using label-free quantification (LFQ) (Fig. 1d)^23,24^. Comparing **pro-N6pA** labelling (Fig. 1e) with parent *N*^6^-propargyl adenosine (**N6pA**, Fig. 1f, Supplementary Fig. 3) yielded a larger number of significantly enriched proteins with the pronucleotide. Using **pro-N6pA**, a diverse group of 19 proteins was identified in HeLa cells, including the known FICD substrate HSPA5 (Fig. 1e, Supplementary Data 1). Immunoprecipitation of the two selected proteins PFKP and PPME1 from the probe treated HeLa cells followed by click reaction with rhodamine-azide tag confirmed incorporation of the probe into these proteins (Supplementary Fig. 3).

Although the *N*^6^-propargyl ATP analogue could, in principle, serve as precursor for ADP-ribosylation^25^, our controls indicate that ADP-ribosylation is not a major route. ADP-ribosylation is usually induced by stress conditions e.g., by addition of hydrogen peroxide to the cells’ media^26^. First, HeLa cells were pre-treated with poly(ADP-ribose)polymerases (PARP) inhibitors 4-aminobenzamide (4-ABA) or olaparib (Ola) prior to **pro-N6pA** labelling. For both PARP inhibitors, no influence on labelling intensity was observed based on in-gel fluorescence analysis (Supplementary Fig. 3). In addition, MS-based chemical-proteomic experiments with Ola and **pro-N6pA** treated cells confirmed no changes in AMPylation (Fig. 1g and Supplementary Data 2). Second, only two of our identified AMPylated proteins in HeLa cells (HIST1H2AH, RPS10) matched known ADP-ribosylated proteins (Supplementary Fig. 3)^27^. Known ADP-ribosylated proteins were excluded as potential hits in the following experiments (Supplementary Data 3).

### AMPylation of cathepsin B inhibits its peptidase activity

**Fig. 2:**
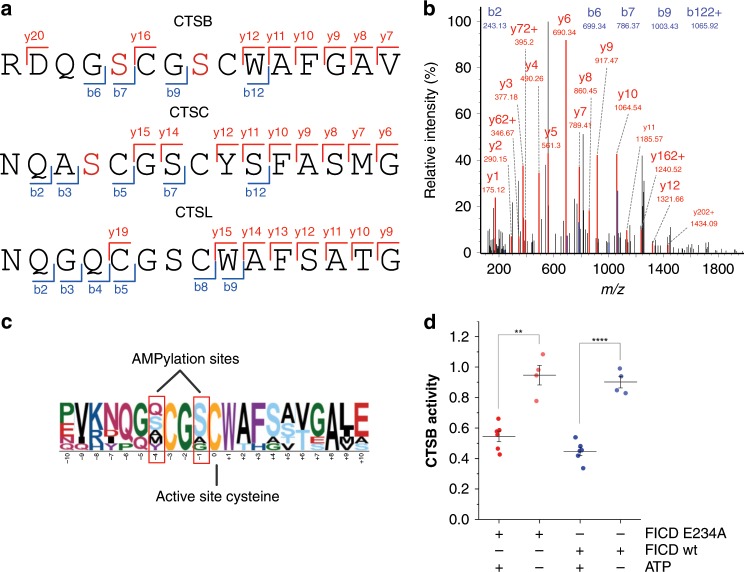
CTSB peptidase activity is inhibited by FICD catalysed AMPylation. **a** Identified AMPylation sites on serine residues (red) of cathepsins B, C and L using **pro-N6pA** in HeLa cells. **b** Exemplary MS/MS spectrum (MaxQuant) for the CTSB AMPylation site identification on S107 (see Supplementary Figs. 4 and 5). **c** Amino acid motif surrounding the active site cysteine of cysteine cathepsins. **d** In vitro peptidase assay of CTSB activity after incubation with wt FICD or E234A mutant and with or without ATP for 6 h. Normalised to CTSB activity without FICD protein. Lines represent the mean and whiskers stand for 25th and 75th percentile. Two-tailed Student’s *t*-test; **P < 0.01, ****P < 0.0001.

In order to validate our approach in more detail, we have employed an azide-TEV-cleavable-biotin linker during the pull-down procedure to identify the corresponding AMPylation sites of modification via MS/MS (Fig. 2a, b)^28^. We were able to directly analyse AMPylated peptides on three different cysteine cathepsin proteases CTSB (S104 and S107), CTSC (S254) and CTSL (S137) in HeLa cells (Supplementary Figs. 4 and 5). All of the AMPylation sites were located on serine residues within the conserved sequence surrounding the catalytically active cysteine (Fig. 2c), suggesting that the bulky AMP modification might obstruct the binding of the peptide substrates and thus inhibit protease activity^29^. To determine whether FICD is the AMPylator of these cathepsins, we used an in vitro peptidase activity assay and found that cathepsin B is indeed inhibited upon FICD (wild-type (wt) or E234A mutant) treatment and did not observe any inhibition without the addition of ATP (Fig. 2d, Supplementary Fig. 4). The direct measurement of AMPylation sites in CTSB (S104,107) in vitro was restricted by preparation of the recombinant double mutant CTSB which did not fold into the active protein, likely due to the mutation of the crucial amino acid residues within the conserved active site. Moreover, the TEV-linker based enrichment of modified peptides was performed with other cell types used in this study and three additional sites on MYH9, RAI14 and AASS (on Thr, Ser, and Tyr residues respectively) were detected (Supplementary Data 4). Of note, the MS-based identification of AMPylated sites in living cells is limited by the endogenous degree of modification. We thus assume that site identifications of proteins with lower AMP abundance are challenged by the detection limit. Here, previous trials in cell lysates using an active recombinant FICD E234G mutant yielded a complementary set of proteins likely due to an increased degree of modification (Supplementary Fig. 6 and Supplementary Data 5)^20^.

### AMPylation profiling shows cell type dependent pattern

**Fig. 3:**
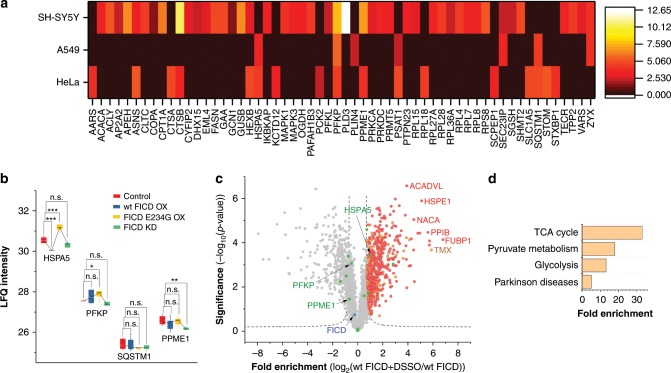
AMPylation in cancer cell lines. **a** Heatmap representation of enriched proteins identified in cancer cell lines. Colour represents distance to zero of enriched proteins from respective volcano plot (FDR 0.05, s0 0.3; *n* = 8 or 9). **b** FICD-dependent AMPylation. Changes in AMPylation on selected proteins (those identified in HeLa cells from Fig. 1e) upon FICD overexpression (OX) or siRNA-mediated knock-down in (KD) HeLa cells. Statistical significance was tested using two-tailed Student’s *t*-test; *P < 0.05 **P < 0.01,***P < 0.001. **c** FICD–interacting proteins. Volcano plot representing FICD interacting proteins identified in the pull-down experiment of his tag labelled FICD and DSSO cross-linking reagent (FDR 0.01; s0 1.5; *n* = 3). Green circles represent proteins identified as AMPylated in HeLa cells. Red circles represent hits overlapping with parallel experiment with FICD E234G mutant. Orange circles are hits enriched only with wt FICD. **d** Panther Pathways enriched from FICD-interacting proteins, cut-off FDR 0.05.

We performed chemical-proteomic profiling in three different cancer cell lines, HeLa, A549 and SH-SY5Y, which revealed a total of 58 significantly enriched proteins, of which 38 were contributed solely from the latter neuroblastoma cells (Fig. 3a, Supplementary Fig. 7). Overall, AMPylated proteins identified here are involved in diverse metabolic pathways including a widely conserved key regulator of glycolysis ATP-dependent 6-phosphofructokinase (PFKP)^30^, proteolysis (CTSA, CTSB)^31^, regulation of PTMs (PPME1)^32^ and UPR (HSPA5 and SQSTM1)^33^. Intriguingly, only PFKP was found to be AMPylated in all three cell lines, which otherwise exhibited unique AMPylation patterns.

To directly dissect the descent of AMPylated proteins from FICD, we compared the AMPylation levels of proteins in probe treated HeLa cells comprising FICD knockdown, wt FICD overexpression (OX) and activated FICD E234G OX (Fig. 3b, Supplementary Fig. 8 and Supplementary Data 6). Interestingly, HSPA5 is a clear FICD-dependent responder where AMPylation is significantly upregulated in FICD E234G OX and downregulated in wt FICD OX, which is also known to perform de-AMPylation^6^. Remarkably, while all previous studies have been carried out in vitro^18–20^, we here independently confirm this data by the first in situ experiments. A direct in situ interaction is further corroborated by MS-based pulldown experiments of wt FICD and FICD E234G in the presence of a chemical crosslinker, which revealed HSPA5 together with other sets of proteins as interacting partners, while proteins like SQSTM1, PFKP and PPME1 were not enriched and thus considered as not interacting with FICD (Fig. 3c)^34^. Of note, FICD E234G revealed a more pronounced interaction with HSPA5, confirming our OX studies (Supplementary Fig. 9 and Supplementary Data 7). GO term analysis of the overlapping interacting partners indicated a link to basal metabolism (Fig. 3d). With HSPA5 as a validated candidate, we moved on and analysed other AMPylated proteins. The set of hits including PPME1, PFKP and SQSTM1 exhibited no significant changes in AMPylation levels upon FICD KD and OX, suggesting an FICD-independent mode of AMPylation (Fig. 3b) for which the origin of the AMP transfer could not be fully deduced. Given the recent discovery of an additional AMPylating enzyme also in eukaryotic cells, it is likely that the other proteins detected here descent form (a) yet undiscovered AMPylator(s)^17^.

Next, chemical-proteomic profiling under endoplasmic reticulum (ER) stress conditions was performed to determine whether modifications are altered as previously reported for thapsigargin (Tg)-treated cells^6^. Only a slight increase by 2.5-fold in AMPylated HSPA5 was observed in HeLa and A549 cells. Quantification of HSPA5 by western blot shows an increase in HSPA5 expression by more than 11-fold after Tg treatment. Thus, normalisation of total AMPylation to the expression of HSPA5 results in an overall reduction of its AMPylation, which is in line with previously published results (Supplementary Fig. 7)^6^. Despite the moderate impact of ER stress on AMPylation in these cells, we found 145 dysregulated proteins in SH-SY5Y neuroblastoma cells (Supplementary Fig. 7). The high amount of AMPylated proteins in SH-SY5Y under baseline and ER stress conditions indicates that AMPylation may have a specific role in the nervous system.

### AMPylation remodels during neuronal differentiation

**Fig. 4:**
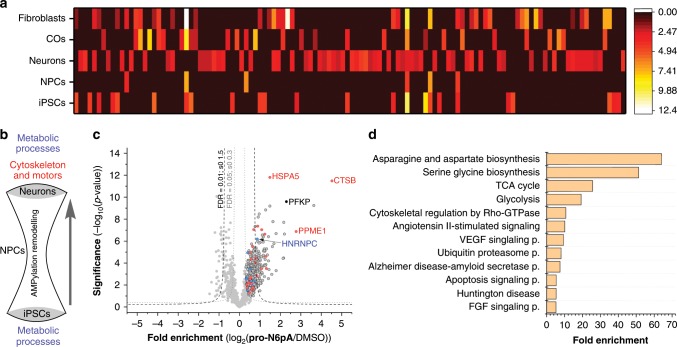
AMPylation remodelling is specific for the development of the neuronal cells. **a** Heatmap representation of enriched proteins identified in different cell types and COs. Colour represents distance to zero of enriched proteins from respective volcano plot (FDR 0.05, s0 0.3; *n* = 8 or 9). **b** The hourglass model of AMPylation remodelling hypothesises a complete de- and re-AMPylation in the process of neuronal differentiation: from a high number of AMPylated proteins in proliferative iPSCs, most of which are involved in metabolic processes and possess catalytic activity, differentiating cells pass through a state of very sparse AMPylation as NPCs, with final neuronal differentiation resulting in neuronal identity with a high number of newly and uniquely AMPylated proteins which are enriched in metabolic functions on the one hand and in cytoskeletal and molecular motor functions on the other hand. **c** Volcano plot of fold-enrichment by **pro-N6pA** labelling compared to DMSO versus significance upon two-sample *t*-test (FDR 0.05, s0 0.3; *n* = 9) in fibroblasts. Red circles represent proteins identified AMPylated in proliferating cell types while blue circles stand for overlap with hits in neurons. **d** Panther Pathways enriched within the identified AMPylated proteins in all tested cell types, cut-off FDR 0.05.

The large number of hits in neuroblastoma cells (Fig. 3a) indicates a specific importance of AMPylation in neural cells. To study AMPylation in a model system of developing neurons, neural progenitor cells (NPCs) and neurons were generated from human induced pluripotent stem cells (iPSCs, Supplementary Fig. 10)^35^. Successively, iPSCs, NPCs and neurons were each treated with **pro-N6pA** and the enriched proteins were analysed via LFQ LC-MS/MS (Fig. 4a, Supplementary Fig. 11 and 12 and Supplementary Data 1 and 8). While PFKP was AMPylated in both the proliferating cell lines and neurons, the proteins CTSB, PSAT1 and PPME1 were only AMPylated in proliferating cells. Importantly, neurons exhibited the largest number of significantly and differentially AMPylated proteins (55 total), including transport proteins (KIF21A, KIF5C, MYH3, MYH7, MYH8) and cytoskeletal proteins (TUBB, TUBB2B, TUBB3B, TUBB4B, MAP2) (Fig. 4a). This is of particular interest as the cytoskeletal remodelling, which is required for neuronal polarisation, migration, and proper axon guidance, is a highly dynamic processes precisely regulated by several PTMs on tubulin and microtubules - and AMPylation may indeed be an additional one (Supplementary Fig. 13)^7,8,36,37^. AMPylation remodelling could be involved in the process of cell type specification and differentiation from iPSCs through NPCs to neurons, with cellular proteins undergoing substantial de- and re-AMPylation following an hourglass-like model (Fig. 4b, Supplementary Fig. 11)^2,6,20^. Further, parallel chemical-proteomics studies of AMPylation under ER stress induced by Tg in iPSCs, NPCs and neurons showed distinct responses ranging from a strong change of AMPylation of several proteins in iPSCs over mild alterations in NPCs to an obvious upregulation on two proteins (HSPA5 and SQSTM1) in neurons (Supplementary Fig. 11).

To specify if the observed AMPylation in neurons is common for differentiated postmitotic cells we performed chemical profiling in fibroblasts (Fig. 4c, Supplementary Fig. 14). Analysis of the enriched proteins revealed similarities with tested cancer cells and proliferating cell types. Most significantly enriched proteins included HSPA5, CTSB, PFKP and PPME1, all common to the proliferating cells. This highlights a distinct AMPylation remodelling in neurons.

GO term analysis of AMPylated proteins found in all screened cell types using the Panther Pathway tool displayed enrichment of basal metabolism such as TCA cycle and glycolysis as well as neuronal specific pathways including cytoskeletal regulation by Rho-GTPase and FGF signalling. Interestingly, pathways marking neurodegenerative diseases, e.g., Alzheimer, the disease-amyloid secretase pathway and Huntingtin disease were identified as well (Fig. 4d).

### FICD and AMPylated proteins have different localisations

**Fig. 5:**
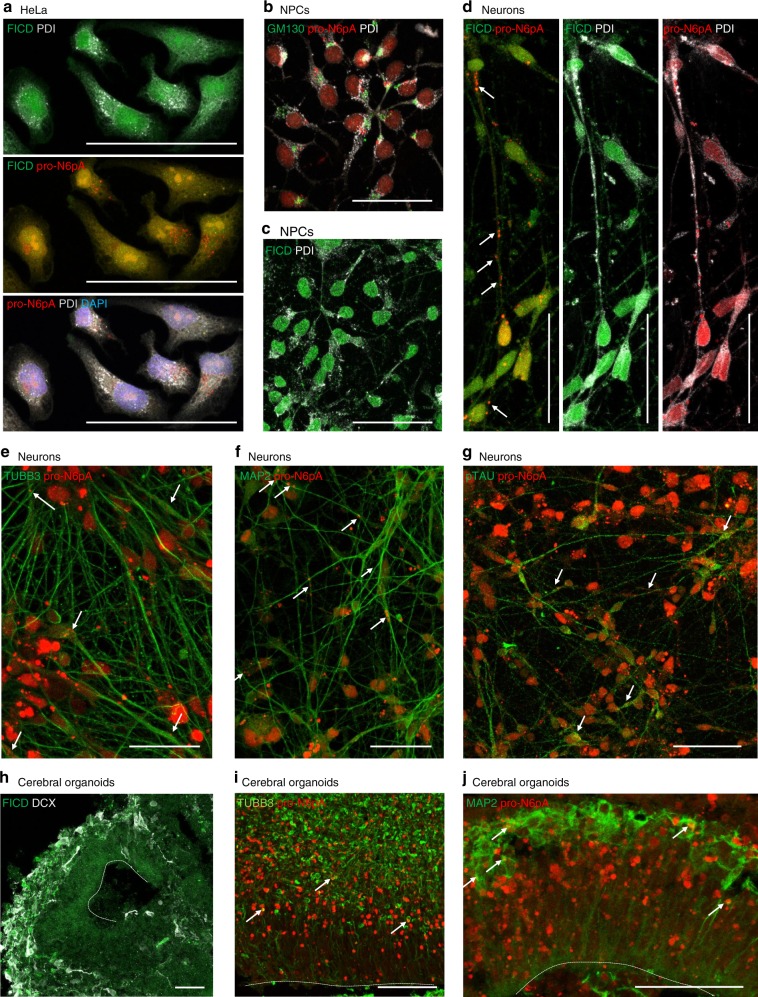
Characterisation of intracellular FICD and probe localisation in HeLa. HeLa **a**, NPCs **b**, **c**, neurons **d**–**g**, and cerebral organoids **h**–**j**. Click chemistry of **pro-N6pA** with rhodamine-azide and immunohistochemical staining. a, HeLa cells contain big nuclear (DAPI – blue) clusters of AMPylated proteins (**pro-N6pA**, red) and additional, small cytoplasmic spots of probe localisation. FICD shows characteristic ER distribution and colocalises with PDI marker, FICD rarely colocalises with probe-containing proteins. **b** In NPCs, probe-containing AMPylated proteins (**pro-N6pA**, red) localise mostly to ER and only rarely with Golgi (GM130, green) with additional small nuclear and cytoplasmic clusters. **c** FICD localises to the ER. **d** In differentiated neurons, clusters of AMPylated proteins localise to nucleus and processes (white arrows), both inside and outside rough ER, partly overlapping with FICD. **e Pro-N6pA** partly colocalises with the TUBB3+ neuronal cytoskeleton (green, white arrows), with TUBB3 being identified as AMPylation target in neurons. **f** AMPylated proteins can be found both in neuronal dendrites (MAP2+ , green, white arrows indicating colocalisation; also identified as neuronal AMPylation target) and **g** in axons (phosphoTAU+ , green, white arrows for colocalisation). **h** In cerebral organoids, FICD (green) is enriched in the DCX+ neuronal layer (white), which is in line with qPCR data from 2D in vitro generated NPCs and neurons (Supplementary Fig. 19). **i, j** AMPylated proteins are enriched right below and within the neuronal layer (TUBB3+ and MAP2+, green) and include TUBB3 (**g**, white arrows indicating colocalisation) and MAP2 (**h**, white arrows indicating colocalisation). Scalebars = 50 µm. See also Supplementary Fig. 15.

The chemical-proteomic results were corroborated by fluorescence imaging of probe-treated HeLa, iPSCs, NPCs and neurons (Fig. 5, Supplementary Fig. 15 and Supplementary Data 9). In order to rule out signals derived from *N*^6^-propargyladenosine nucleotide incorporation into RNAs (e.g., in polyA tails of mRNA)^38^, we performed a control experiment in which the RNA of the fixed cells was digested with different concentrations of RNase prior to click-chemistry and as positive control of the RNase digest 5-ethynyl uridine (5-EU) stained RNAs were degraded in parallel to **pro-N6pA** labelling. Indeed, we observed only a slight decrease in overall cell staining by **pro-N6pA** rather than disappearance of the bright AMPylation spots, while we observed a strong decrease in 5-EU labelling (Supplementary Fig. 16).

Given that the cellular localisation of FICD and AMPylated proteins might play an important functional role, we combined click chemistry with rhodamine-azide for intracellular probe visualisation with immunohistochemistry (IHC) for FICD and various cellular markers: PDI for rough endoplasmic reticulum, GM130 for Golgi complex, TUBB3 for total neuronal microtubule cytoskeleton, MAP2 for neuronal dendrites, phospho-TAU for neuronal axons, and DAPI to visualise nuclei (Fig. 5, Supplementary Fig. 15). Staining performed in HeLa cells revealed that AMPylated proteins are enriched in the nucleus, additional small spots were found in the cytoplasm partially overlapping with the ER. As expected, FICD is localised in the ER (Fig. 5a). This observation was further corroborated by overexpression of FICD with a C-terminal FLAG tag (Supplementary Figure 17) and by analysis of the FICD’s glycosylation using endoglycosidase H assay (Supplementary Fig. 17). On the contrary, in NPCs AMPylation is strictly localised next to the rough ER and in the nucleus (Fig. 5b, c). In neurons, AMPylation was observed in nucleus and to a lesser extent in neurites, including MAP2+ dendrites and phospho-TAU+ axons (Fig. 5d-g). Finally, fibroblasts showed another specific localisation pattern with AMPylation accumulated around the nucleus and its’ complete absence inside (Supplementary Figure 15). Differences in localisation of FICD and AMPylated proteins support the presence of additional AMP transferases with complementary cellular distribution.

### FICD knockdown reduces neuronal differentiation

To understand if AMPylation plays a role in neuronal differentiation, we utilised both NPC-to-neuron differentiation and the recently developed 3-dimensional human cerebral organoids (COs)^39,40^. COs contain areas which closely resemble the structure and organisation of the germinal zones of developing human neocortex (Supplementary Figure 18)^41^. Treatment of COs with **pro-N6pA** and subsequent analysis via LFQ LC-MS/MS confirmed the AMPylation of PFKP, found in all cell types, and CTSB, another prevalent target in other studied cell lines (Fig. 4a and Supplementary Figs. 5 and 12). Analysis of the significantly enriched proteins using a STRING database revealed that several proteins are located in extracellular space (Supplementary Fig. 13). Interestingly, visualisation of the **pro-N6pA** probe-treated COs via click-chemistry with rhodamine-azide revealed strongest fluorescence in the neuronal layer (Fig. 5i, j and Supplementary Fig. 15), which is in line with the highest number of AMPylated proteins identified in neurons (Fig. 4a).

**Fig. 6:**
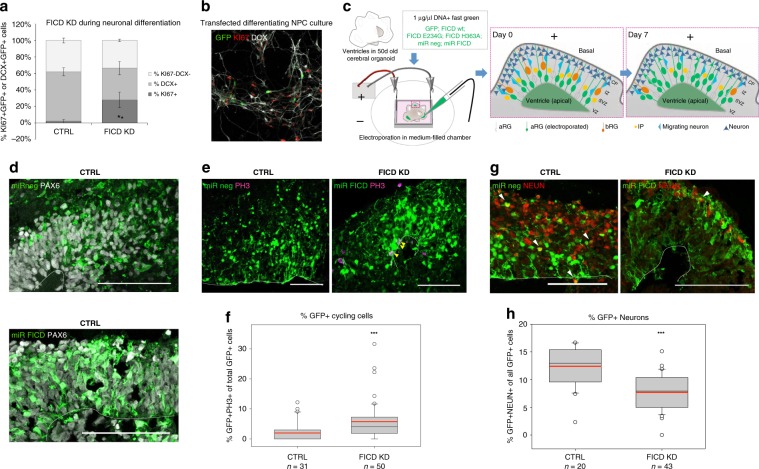
Downregulation of FICD levels keeps differentiating neurons in a cycling state. **a** Quantification of IHC staining for the proliferation marker KI67+ and the early neuronal marker doublecortin (DCX) showed that FICD KD leads to a significant increase in KI67+ compared to control, while the number of generated neurons tends to be decreased (analysis of 3 coverslips/condition with at least 20 transfected cells each; two-tailed Student’s *t-test:* KI67+ : *P < 0.05; DCX+ : *P* *=* *0.068*). **b** Example image of transfected and IHC stained culture with transfected cells (GFP+) in green, proliferating cells (KI67+) in red and differentiating neurons (DCX+) in white. **c** Scheme showing the electroporation of DNA into ventricle-like structures of cerebral organoids (COs) and the organisation of different cell types within the germinal zone. DNA (constructs are listed; supplemented with fast green for visualisation) is injected into the lumen and taken up by aRG via their apical processes. The transfected construct can be found in IPs and neurons upon differentiation of transfected aRG (green, 7 days post electroporation (dpe)) (VZ = ventricular zone, SVZ = subventricular zone, IZ = intermediate zone, CP = cortical plate; aRG = apical radial glia, bRG = basal radial glia, IP = intermediate progenitor). **d** Upon acute miRNA-mediated KD of FICD in ventricles of COs (50d + 7), most GFP+ cells (green) have aRG identity (PAX6+ , white). **e**, **f** FICD KD leads to an increased number of cycling progenitors (**e** IHC staining for PH3+ cells in M-Phase. GFP+ PH3+ cells marked by yellow arrowheads; **f** Quantification of GFP+ PH3+ progenitor cells 7 dpe). **g**, **h** aRG transfected with FICD-targeting miRNAs differentiate less to neurons (**g**, IHC staining for neuronal nuclei marker NEUN, red; GFP-positive neurons shown by white arrowheads; **h**, Quantification of GFP+ neurons 7 dpe shows significant decrease upon FICD knockdown). **d, e, g** 50 + 7d old organoids; electroporated cells and their progeny shown in green; Scalebar = 50 µm, dotted line = apical surface. **f, h** 1*n* = 1 electroporated germinal zone; box plot: mean (red line), median (black line), box represents 25th and 75th percentiles, whiskers extend to 10th and 90th percentiles, all outliers are shown; Significance was tested using Kruskal-Wallis One-way ANOVA on Ranks and Dunn’s Pairwise Multiple Comparison (***P < 0.001).

**Fig. 7:**
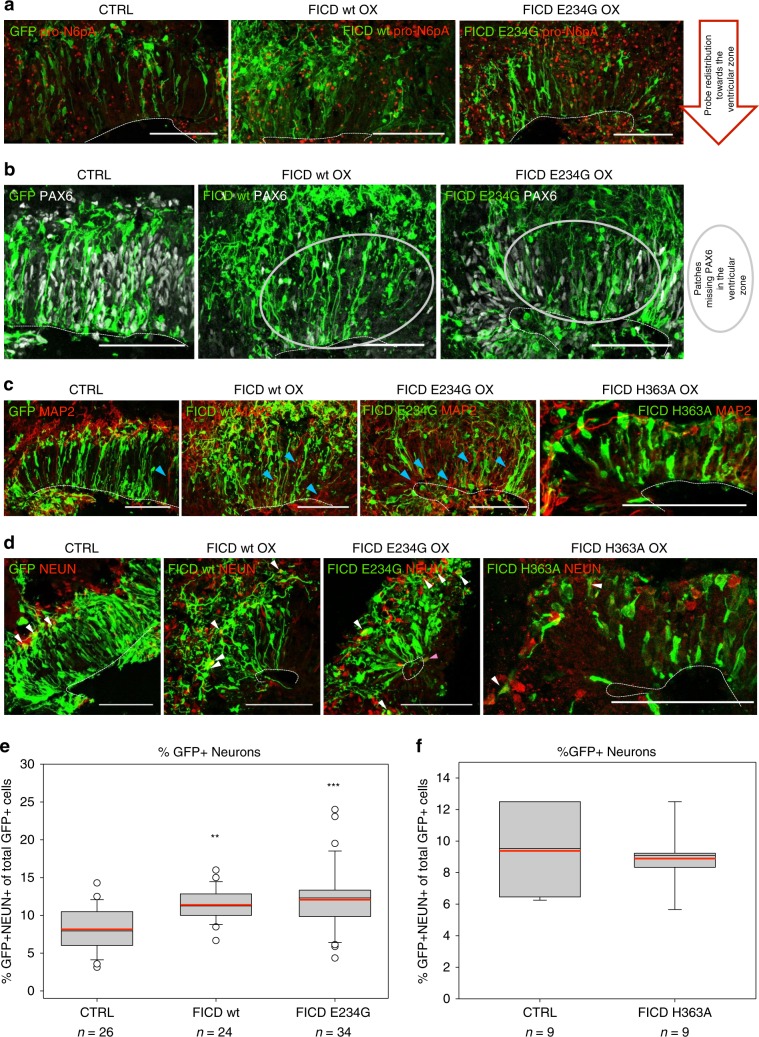
FICD overexpression increases neuronal differentiation in cerebral organoids. FICD wt, E234G and H363A were overexpressed in 50d old cerebral organoids (COs, see Fig. 6c for electroporation scheme) and sections were analysed 7 days later by immunohistochemistry (IHC). FICD constructs do not bear any fluorescent tag and were co-transfected with GFP containing plasmid as a transfection control (green colour). **a** Acute overexpression (OX) of FICD wt and E234G (green) in ventricles of COs (50 + 7d) leads to remodelling of AMPylation, visualised by the redistribution of fluorescence labelling using **pro-N6pA** (red). **b** Germinal zones rich in cells overexpressing FICD wt or E234G (green) show patchy “holes” lacking PAX6+ dorsal NPCs (grey) in their ventricular zone (VZ) (indicated by grey circle). **c** Upon FICD wt and E234G OX, MAP2+ neuronal processes (red) increasingly extend into the VZ (blue arrowheads), which does not occur upon control and FICD H363A OX. **d**, **e** RGs overexpressing FICD wt or E234G show increased differentiation to neurons compared to control. (**d** IHC staining for nuclei of differentiated neurons (NEUN, red; GFP+ neurons shown by white arrowheads, GFP+ neuron in the progenitor zone by blue arrowhead. **e** Quantification of GFP+ neurons shows significant increase upon FICD wt or E234G OX). **f** OX of the catalytically inactive FICD H363A does not lead to an increase in GFP+ neurons. **a**, **b**, **c**, **d** 50 + 7d old organoids; electroporated cells and their progeny shown in green; Scalebar = 50 µm, dotted line = apical surface. **e**, **f** 1*n* = 1 electroporated germinal zone; box plot: mean (red line), median (black line), box represents 25th and 75th percentiles, whiskers extend to 10th and 90th percentiles, all outliers are shown; significance was tested using Kruskal–Wallis One-way ANOVA on Ranks and Dunn’s Pairwise Multiple Comparison (**P < 0.01; ***P < 0.001). See also Supplementary Fig. 21 for analysis of PH3+ progenitors upon FICD wt/E234G/H363A OX in COs and for scoring of MAP2+ progenitor cells intruding the VZ upon FICD KD or FICD wt/E234G/H363A OX in COs and Supplementary Fig. 22 for the analysis of 2 weeks after electroporation of COs with FICD wt/E234G OX constructs.

To examine the function of AMPylation in neurogenesis and neuronal differentiation in more detail, we first characterised the expression of FICD in NPCs, neurons, neuroblastoma cells and COs and found a clear enrichment of FICD in the neurites of neurons, SH-SY5Y and in the neuronal layer of COs compared to the progenitor zone (Fig. 5d, h; Supplementary Fig. 15). Results of imaging were paralleled by qPCR studies demonstrating higher baseline expression levels of FICD in neurons compared to iPSCs and NPCs (Supplementary Fig. 19). We knocked down FICD levels (Supplementary Fig. 20) in NPCs differentiating to neurons (Fig. 6a, b) and found a significant increase in transfected cells that remain in cell cycle (KI67+) (Fig. 6a). This result suggested a potential role of FICD-mediated AMPylation in neurogenesis. We then performed down- or upregulation of FICD expression in ventricle-like germinal zones of 50 days old COs by electroporation, as this model system better resembles the 3-dimensional organisation of the developing brain. Only apical radial glia cells (aRGs), which are bipolar neural stem cells that will subsequently give rise to intermediate progenitors and neurons directly, are capable of taking up the vectors via their apical process to the ventricle-like lumen (Fig. 6c). To asses if FICD-mediated activity has a function in neurogenesis during development, COs were analysed 7 (Figs. 6 and 7, Supplementary Fig. 21) and 14 days post-electroporation (dpe) (Supplementary Fig. 22). Cortical-like germinal zones were defined by immunohistochemical (IHC) analysis using PAX6 as a marker for dorsal aRGs (Figs. 6d, and  7b) with mitotic cells labelled for PH3 (Fig. 6e, Supplementary Fig. 21). The position and number of neurons was analysed by IHC using two different markers for mature neurons: MAP2, a microtubule-associated protein which is enriched in neuronal dendrites (Fig. 7c, Supplementary Fig. 21) and the nuclear marker NEUN (Figs. 6g and  7d). Most of miRNA-transfected (GFP+) cells (FICD KD) were positive for PAX6 (Fig. 6d) 7 dpe. The proportion of mitotic PH3+ GFP+ cells was significantly increased (Fig. 6e, f) at the expense of neurons, as shown by the significantly reduced number of NEUN+ GFP+ cells (Fig. 6g, h).

### FICD overexpression increases neuronal differentiation

Conversely, when electroporating vectors carrying wt FICD, activated FICD E234G mutant or catalytically inactive FICD H363A mutant into ventricles of 50 days old COs, those transfected with FICD wt or E234G showed an increase and redistribution in fluorescent signal upon **pro-N6pA** treatment indicating a remodelling of AMPylation upon FICD overexpression (Fig. 7a), while there were no changes in distribution or intensity of the signal upon OX of FICD H363A used as a control (Supplementary Fig. 21 and similarly in neuroblastoma cells Supplementary Fig. 23). Moreover, upon FICD wt or E234G OX in COs, progenitor zones had regions sparse in PAX6+ cells (Fig. 7b). At the same time, MAP2+ neurites increasingly invaded these progenitor zones 7 dpe (Fig. 7c, blue arrowheads; Supplementary Fig. 21) and 14 dpe (Supplementary Fig. 22), which was not the case upon control or FICD H363A electroporation (Fig. 7c), nor upon FICD KD (Supplementary Fig. 21). Interestingly, both the E234G mutant and wt FICD-transfected aRGs gave rise to a significantly higher number of neurons compared to H363A inactive mutant or control already at 7 dpe (Fig. 7e, f), which was consistent also at 14 dpe (Supplementary Fig. 22). Additionally, we have excluded other cellular processes to be involved in the observed effect by whole proteome analysis of FICD transfected neuroblastoma cells (Supplementary Fig. 24, Supplementary Data 10), which showed only minimal changes in overall protein expression. Furthermore, pull-down of the AMPylated proteins from neuroblastoma cells under FICD E234G OX conditions revealed a general increase in AMPylation including proteins such as CTSB, TPP1, CAPZB, and NSFL1C, which were found AMPylated in COs (Supplementary Fig. 25, Supplementary Data 11). This effect was not observed with FICD wt OX, which is in line with low AMPylation activity of the FICD wt.

Taken together, FICD may regulate the transition from neural progenitors to neurons. The direct comparison to catalytically inactive FICD H363A, showing no difference to control condition, demonstrates the importance of FICD catalytic activity in proper progenitor cell cycle exit and neuronal differentiation. These results suggest that remodelling of AMPylation may play a role in neuronal differentiation during human brain development. However, it remains to be investigated whether the specific AMPylation/de-AMPylation activity on HSPA5 and subsequent changes in the UPR are responsible for modulation of the neuronal differentiation, which would be supported by the known connection between UPR and brain development^42^. Alternatively, the synergistic action of AMPylation on cytoskeletal protein targets catalysed by putative AMPylators and associated changes in cellular polarisation as described for example for MAP6 palmitoylation could be responsible for these effects^43^.

## Discussion

Our **pro-N6pA** phosphoramidate probe design facilitated in situ identification of 162 potentially AMPylated proteins in different cell types and uncovered FICD as a modulator of neuronal differentiation. We successfully identified FICD dependent AMPylation as exemplified on HSPA5 and FICD independent AMPylation as shown for other proteins like PFKP, PPME and SQSTM1. FICD is the only known human AMPylator and all previous studies utilise this enzyme for deciphering substrates in vitro. Our in situ approach is global and does not only depend on FICD. Thus FICD-independent AMPylation supports the existence of additional AMP transferases such as an emerging group of pseudokinases which were identified as AMPylators in eukaryotic cells^17^. Moreover, our in situ profiling allowed to screen AMPylation remodelling during neuronal development from iPSCs in 2D and 3D in vitro approaches, connecting biological implications of FICD dependent AMPylation/de-AMPylation with human brain development: Acute KD of FICD in differentiating neurons (2D) and in aRG in COs (3D) kept cells in a cycling state, while OX of the only known human AMPylating enzyme was shown to drive the differentiation of NPCs to neurons in COs. The subtle but always significant dysregulation of neurogenesis resulting from FICD OX and KD may be caused by impaired AMPylation remodelling, influencing catalytic activity of metabolic enzymes or stability of cytoskeletal proteins. The remarkable number of AMPylated targets identified altogether in NPCs, neurons and COs indicates a synergistic influence in fine-tuning neurogenesis, but it is not trivial to pinpoint the function of each target protein individually, leaving the precise molecular mechanism unresolved. Furthermore, alteration of the neuronal differentiation process might be influenced as well through the AMPylation of HSPA5 and successive changes in UPR^16^.

Our study highlights both the promises and challenges of using chemical-proteomics for identification of protein PTMs. Although we have successfully identified a large group of AMPylated proteins in various cell types and elucidated its functional implications, the method itself yielded rather low rate of identified sites needed for biochemical testing of the AMPylation function in vitro. Nevertheless, we were able to identify seven sites and show that this PTM can inhibit target protein activity, as exemplified by CTSB, the abundance of AMPylation likely limits in situ detection. Future studies will thus focus on methods to quantify AMPylation levels and fine-tune enrichment and MS-based detection procedures. Interestingly, taking together both approaches of chemical-proteomics and fluorescence imaging utilising the **pro-N6pA** probe suggests a cell type-specific AMPylation pattern. This is a combination of the particular AMPylated proteins and their intracellular localisation in a certain cell type. For example, postmitotic fibroblasts exhibit highly enriched proteins shared with the proliferating cell lines, but their subcellular localisation is very distinct from the localisation in the cycling cells. Aside the dependence of AMPylation on the cell type, we have shown with the example of thapsigargin-induced ER stress that the prevalent environmental condition can affect AMPylation.

With these features, we believe our method will lead to discovery of new functions for protein AMPylation beyond neuronal development e.g., in stem cell differentiation, unfolded protein response or regulation of complex network of cysteine cathepsins.

## Methods

### Synthesis

Synthesis of the phosphoramidate probe **pro-N6pA** is described in the Supplementary Information^44,45^. Chemical identity and samples purity were established using NMR, HRMS and HPLC analysis.

### Cell lines

Human epitheloid cervix carcinoma cells (HeLa, CCL-2) and human lung carcinoma cells (A549) were cultivated in high glucose Dulbeccos´s Modified Eagle´s Medium (DMEM) supplemented with 10% (v/v) fetal bovine serum (FBS) and 2 mM l-glutamine. Cells were grown under a humidified atmosphere at 37 °C and 5% CO_2_. Cells were seeded into 6 cm diameter dishes and grown to 80–90% confluency. Human neuroblastoma cells SH-SY5Y (CRL-226) were cultivated in DMEM/F12 1:1 media supplemented with 10% (v/v) FBS.

### Chemical-proteomics

Cells were treated with the probes at 80–90% confluency (*n* represents number of cell culture dishes). Culture medium was removed and the cells or COs were labelled in fresh media containing 100 µM **N6pA** or 100 µM **pro-N6pA** (both stocks 100 mM in DMSO) for 16 h at 37 °C in cells incubator. Subsequent cell lysis, click chemistry, avidin beads enrichment and MS sample preparation were performed as described in Supplementary Information and literature^22–24,46^. A total amount of 500 µg (HeLa, A549, SH-SY5Y or COs) or 250 µg (iPSCs, NPCs, neurons) of proteins in lysate was used for each MS sample preparation. For details see Supplementary Information.

### Site identifications

Site identification experiments were performed in HeLa and SH-SY5Y cells and COs. Cells or COs were cultivated and treated with **pro-N6pA**. After the cells’ lysis and protein concentration measurement, 3.6 and 16 mg of HeLa or 6 mg of SH-SY5Y or 8 mg of CO protein lysates were used for further MS sample preparations. The protocol used for enrichment and digest with TEV-cleavable linker was adapted from ref. ^28^. For details see Supplementary Information.

### Mass Spectrometry

Nanoflow LC–MS/MS analysis was performed with an UltiMate 3000 Nano HPLC system coupled to an Orbitrap Fusion or Q Exactive Plus (Thermo Fisher Scientific). Fragments were generated using higher-energy collisional dissociation (HCD) and detected in the ion trap at a rapid scan rate. Raw files were analysed using MaxQuant software with the Andromeda search engine. Searches were performed against the Uniprot database for Homo sapiens (taxon identifier: 9606, 7th July 2015, including isoforms). At least two unique peptides were required for protein identification. False discovery rate determination was carried out using a decoy database and thresholds were set to 1% FDR both at peptide-spectrum match and at protein levels. For AMPylation site identification spectra were searched for AMP conjugated with TEV tag (+ 694.2700) and only one unique or razor peptide was required. For details of MS measurement and data analysis see Supplementary Information and Supplementary Data 1, 4 and 8.

### In vitro CTSB activity assay

Cathepsin B (CTSB, 0.4 μg/μL, R&D Systems) was diluted in activation buffer (25 mM MES, 5 mM DTT, pH 5.0) to 10 μg/mL and incubated at 25 °C for 25 min. The activated CTSB was further diluted to 2 μg/mL in AMPylation buffer (20 mM Hepes, 100 mM NaCl, 5 mM MgCl_2_, 1 mM DTT, 0.1 mg/mL BSA, pH 7.5) and supplemented with 100 μM ATP, 2.8 μM wt FICD or FICD E234G mutant (gift from A. Itzen, TUM) or ddH_2_O and incubated at 25 °C for 0–6 h. Subsequently, 3 μL of the mixture was used in 57 μL assay buffer (25 mM MES, 10 μM Z-Arg-Arg-7-amido-4-methylcoumarin hydrochloride (Sigma), pH 5.0) in 96-well plate and the fluorescent intensity was read by TECAN 200 M Pro after 20 min using 380 nm and 460 nm as excitation and emission wavelengths.

### iPSC culture

Induced pluripotent stem cells reprogrammed from fibroblasts (for reprogramming see Supplementary Information) were cultured at 37 °C, 5% CO_2_ and ambient oxygen level on Geltrex coated plates (Thermo Fisher Scientific) in mTeSR1 medium (StemCell Technologies) with daily medium change. For passaging, iPSC colonies were washed with PBS and incubated with StemPro Accutase Cell Dissociation Reagent (A1110501, Life Technologies) diluted 1:4 in PBS for 3 min. Pieces of colonies were washed off with DMEM/F12, collected by 5 min centrifugation at 300 × *g* and resuspended in mTeSR1 supplemented with 10 µM Rock inhibitor Y-27632(2HCl) (72304, StemCell Technologies) for the first day.

### Generation of neural progenitor cells (NPCs) and neurons

Neural progenitors were generated according to the literature procedures^35^ with the following modifications. In brief, embryoid bodies (EBs) were generated from feeder-free iPSCs by incubating colonies with Collagenase Type IV (7909, StemCell Technologies) for 10 min, followed by washing with DMEM/F12, manual disruption and scraping with a cell lifter (3008, Corning Life Sciences). Resulting pieces of colonies were plated in suspension in Neural Induction Medium (NIM) consisting of DMEM/F12 + Hepes (31330095, Life Technologies) with 1× N2 and B27 supplements (without vitamin A, Thermo Fisher) with medium change every other day. Resulting NPCs were passaged using Accutase (StemCell Technologies) and split at a maximum ratio of 1:4. NPCs were only used for up to seven passages. For differentiation to neurons, single NPCs were plated at a density of 10^4^ cells/cm^2^ on Polyornithine/Laminin plates and cultured in NPM for 1 more day to reach about 30% cell density. Afterwards, medium was changed to Neuronal Differentiation Medium NDM (NIM containing 20 ng/mL BDNF (248-BD, R&D Systems) and 20 ng/mL GDNF (212-GD, R&D Systems)) and cells were differentiated for 40 days with medium change every 5 days.

### Cerebral organoids

Cerebral organoids were generated starting from 9000 single iPS cells/well^40^. Organoids were cultured in 10 cm dishes on an orbital shaker at 37 °C, 5% CO_2_ and ambient oxygen level with medium changes twice a week. Organoids were electroporated at 50 days after plating (see Electroporation of cerebral organoids) and analysed 7 and 14 dpe. For immunostaining, 16 µm sections of organoids were prepared using a cryotome. For analysis 7 dpe, 24–34 different ventricles in 7–12 organoids from 2 independent batches were analysed per construct. For 14 days, 4 organoids per construct with altogether 13–21 electroporated ventricles per construct were analysed.

### Generation and validation of microRNAs targeting FICD

MicroRNAs (miRNAs, Table 1) targeting FICD were generated using the BLOCK-iT system from Invitrogen (Thermo Fisher, Waltham, MA, USA). MiRNA sequences were determined using Invitrogens RNAi design tool https://rnaidesigner.thermofisher.com/rnaiexpress/setOption.do?designOption = mirnapid = 1961720787891316464, accessed on December 6th, 2017, with the NCBI Reference Sequence NM_007076.2 as seed sequence. Three miRNA sequences were chosen and ordered as oligonucleotides from Sigma. FICD miRNA oligonucleotides were annealed and ligated into a GFP-containing entry vector pENTR-GW/EmGFP-miR using T4 DNA Ligase (Thermo Fisher, Waltham, MA, USA). Subsequently, the miRNA sequences were cloned into the pCAG-GS destination vector using the Gateway system (Thermo Fisher). The resulting miRNA expression plasmids were sequenced, the knockdown efficiency was validated in Hela cells via qPCR and Westernblot and the most efficient construct was used for NPC transfection and for electroporation of COs (Fig. 6, Supplementary Fig. 19).

**Table 1:** miRNA sequences used in the study.

Oligo Name	Sequence (5′ to 3′)
miRNA FICD_top	TGCTGAATGCTCTTCCACAACTCCCAGTTTTGGCCACTGACTGACTGGGAGTTGGAAGAGCATT
miRNA FICD_bottom	CCTGAATGCTCTTCCAACTCCCAGTCAGTCAGTGGCCAAAACTGGGAGTTGTGGAAGAGCATTC

### Transfection of differentiating NPCs

For transfection of differentiating NPCs, 10^4^ cells/cm^2^ were plated on Polyornithine/Laminin-coated coverslips in 24-well plates. After one day in NPM (see Generation of neural progenitor cells (NPCs) and neurons from iPSCs), medium was changed to growth-factor free NIM (see Generation of neural progenitor cells (NPCs) and neurons from iPSCs.) to generate differentiating conditions. 4 days after plating, NPCs were transfected with 500 ng DNA/well following Lipofectamine® 3000 protocol (Thermofisher) and continuously cultured in NIM with medium change every other day. Cells were fixed 7 days post transfection with 4% PFA for 20 min at RT and processed by immunohistochemistry.

### Electroporation of cerebral organoids

For electroporation (see scheme in Fig. 6c), cerebral organoids were kept in NDM + A without antibiotics. The organoids were placed in an electroporation chamber (Harvard Apparatus) and pCMV-SPORT6 plasmid with FICD wt, FICD E234G (gift from A. Itzen, TUM), or FICD H363A plus pCAG-IRES-GFP (FICD to GFP ratio 2:1), GFP only as overexpression control, miRNA against FICD (or scrambled miRNA negative control) in pCAG-GS at a concentration of 1 µg/µl, supplemented with fast green for visualisation, was injected into ventricle-like cavities at several positions per organoid. Electroporation was performed with an ECM830 electroporation device (Harvard Apparatus) by subjecting the organoids to a 1 s interval with 5 pulses of 50 ms duration at 80 mV.

### Immunohistochemistry

Frozen organoid sections were thawn to rt for 20 min and then rehydrated in PBS for 5 min. For nuclear antigens, an antigen retrieval step (HIER) was performed in which the sections were boiled in 0.01 M citric buffer pH 6 for 1 min at 720 Watt and an additional 10 min at 120 W. Slides were then left to cool down for 20 min. Half of the citric buffer was replaced by H_2_O, slides were incubated for another 10 min and then washed in PBS. Subsequently, a postfixation step of 10 min was carried out with 4% PFA in PBS. Then, the sections were permeabilized using 0.1% Triton X100 in PBS for 5 min. After permeabilization, sections were blocked at rt for at least 1 h with 10% Normal Goat Serum in 0.1% Tween in PBS. The primary antibody (Supplementary Table 1 in Supplementary Information) in blocking solution was then incubated overnight at 4 °C. Following several washes with 0.1% Tween in PBS, sections were incubated with 1:1000 dilutions of Alexa Fluor-conjugated secondary antibodies (Life Technologies) in blocking solution for at least 1 h at rt, using 0.1 µg/ml 4,6-diamidino-2-phenylindole (DAPI, Sigma Aldrich) to counterstain nuclei. Finally, sections were washed again several times with 0.1% Tween in PBS and mounted with Aqua Polymount (18606, Polysciences). Sections were visualised using a Leica SP8 confocal laser scanning microscope. Cells were cultured on round coverslips (13 mm diameter, VWR) in 24 well plates, washed with PBS and fixed with 4% PFA in PBS for 15 min at rt. HIER, permeabilization, blocking and staining were carried out as described for the organoid sections.

### Cell quantifications

For quantification of GFP^+^ mitotic cells or neurons upon NPC transfection or in electroporated CO ventricles, all GFP^+^PH3^+^, GFP^+^KI67^+^, GFP^+^DCX^+^, and GFP^+^NEUN^+^ cells were counted using the cell counter plugin in Fiji^47^. Double positive cells were normalised to the total number of GFP^+^ cells.

### Statistics

Statistical analysis of the MaxQuant result table proteinGroups.txt (Supplementary Data 2) was done with Perseus 1.5.1.6. Putative contaminants and reverse hits were removed. Dimethyl-labelling ratios or normalised LFQ intensities were log_2_-transformed, hits with <3 valid values in each group were removed and −log_10_(*p*-values) were obtained by a two-sided one sample Student’s *t*-test over replicates with the initial significance level of *p* = 0.05 adjustment by the multiple testing correction method of Benjamini and Hochberg (FDR = 0.05), the −log_10_ of *p*-values were plotted against the log_2_ of geometric mean of ratios “heavy”/”light” (H/L) for dimethyl labelling or by volcano plot function for LFQ. Distance from zero was calculated from significance and fold enrichments from respective volcano plot as d=√((*fold encrichment*)^2^+(*significance*)^2^) Venn diagrams were generated with a drawing tool at http://bioinfogp.cnb.csic.es/tools/venny/ using gene names as a key. All graphs were processed in Microsoft Excel or OriginPro 2017. Statistics for qPCR data and quantifications of immunohistochemical stainings in cells and COs was performed in SigmaPlot (Version 13.0; Systat Software, San Jose, CA) using Kruskal-–Wallis ANOVA on Ranks with Dunn’s Pairwise Multiple Comparison. For NPC transfection, 3 coverslips with at least 20 transfected cells each were analysed. For COs, 2–4 batches of organoids were analysed for each construct (Data shown with *n* = total number of electroporated ventricles analysed per construct).

### Reporting summary

Further information on research design is available in the Nature Research Reporting Summary linked to this article.

## Supplementary information

Supplementary Information

Reporting Summary

Description of Additional Supplementary Files

Supplementary Data 1

Supplementary Data 2

Supplementary Data 3

Supplementary Data 4

Supplementary Data 5

Supplementary Data 6

Supplementary Data 7

Supplementary Data 8

Supplementary Data 9

Supplementary Data 10

Supplementary Data 11

Supplementary Data 12

## Data Availability

The mass spectrometry proteomics data have been deposited to the ProteomeXchange Consortium via the PRIDE partner repository with the dataset identifier PXD015062 (http://www.ebi.ac.uk/pride/archive/projects/PXD015062).
