# Supplementary material for: FICD activity and AMPylation remodelling modulate human neurogenesis
Source: Nat Commun. 2020 Jan 24;11:517. doi: 10.1038/s41467-019-14235-6 (PMC6981130; doi:10.1038/s41467-019-14235-6)
Supplement: Supplementary file 2 — Reporting Summary [file 41467_2019_14235_MOESM2_ESM.pdf]

nature research

Corresponding author(s): Prof. Stephan A. Sieber

Life Sciences Reporting Summary

Nature Research wishes to improve the reproducibility of the work that we publish. This form is intended for publication with all accepted life science papers and provides structure for consistency and transparency in reporting. Every life science submission will use this form; some list items might not apply to an individual manuscript, but all fields must be completed for clarity.

For further information on the points included in this form, see Reporting Life Sciences Research. For further information on Nature Research policies, including our data availability policy, see Authors & Referees and the Editorial Policy Checklist.

Please do not complete any field with "not applicable" or n/a. Refer to the help text for what text to use if an item is not relevant to your study. For final submission: please carefully check your responses for accuracy; you will not be able to make changes later.

Experimental design

1. Sample size

Describe how sample size was determined.

The minimal sample size for chemical proteomics was n = 8, supposed effect size d = 2 at alpha error probability = 0.05 and Power = 0.95. For electroporation of cerebral organoids 1) analysis 7 dpe, 24-34 different ventricles in 7-12 organoids from 2 independent batches were analysed per construct. For 14 days, 4 organoids per construct with altogether 13-21 electroporated ventricles per construct were analysed. We see 1 electroporated germinal zone as 1n, so as one independent biological replicate.

2. Data exclusions

Describe any data exclusions.

No data were excluded from the analysis.

3. Replication

Describe the measures taken to verify the reproducibility of the experimental findings.

All attempts of the replication were successful. Findings from the first batch of electroporated organoids were confirmed and thus replicated in the second batch. qPCR: 2 technical replicates were performed and, together with imaging results and the fact that qPCR is a well-established method, we concluded this to be enough. SH-SY5Y transfection: n = 90 GFP+ (=transfected) cells per condition were analysed altogether, originating from 3 biological replicates (3X30 cells).

4. Randomization

Describe how samples/organisms/participants were allocated into experimental groups.

Samples were allocated into groups based on the cell type and preparation.

5. Blinding

Describe whether the investigators were blinded to group allocation during data collection and/or analysis.

Blinding was not possible.

Note: all in vivo studies must report how sample size was determined and whether blinding and randomization were used.

November 2017

6. Statistical parameters

For all figures and tables that use statistical methods, confirm that the following items are present in relevant figure legends (or in the Methods section if additional space is needed).

n/a	Confirmed	
☐	☒	The exact sample size (n) for each experimental group/condition, given as a discrete number and unit of measurement (animals, litters, cultures, etc.)
☐	☒	A description of how samples were collected, noting whether measurements were taken from distinct samples or whether the same sample was measured repeatedly
☐	☒	A statement indicating how many times each experiment was replicated
☐	☒	The statistical test(s) used and whether they are one- or two-sided<br>*Only common tests should be described solely by name; describe more complex techniques in the Methods section.*
☐	☒	A description of any assumptions or corrections, such as an adjustment for multiple comparisons
☐	☒	Test values indicating whether an effect is present<br>*Provide confidence intervals or give results of significance tests (e.g. P values) as exact values whenever appropriate and with effect sizes noted.*
☐	☒	A clear description of statistics including central tendency (e.g. median, mean) and variation (e.g. standard deviation, interquartile range)
☐	☒	Clearly defined error bars in all relevant figure captions (with explicit mention of central tendency and variation)

See the web collection on statistics for biologists for further resources and guidance.

Software

Policy information about availability of computer code

7. Software

Describe the software used to analyze the data in this study.	MaxQuant, Perseus, Fiji, Origin, SigmaPlot, STRING.db, Xcalibur

For manuscripts utilizing custom algorithms or software that are central to the paper but not yet described in the published literature, software must be made available to editors and reviewers upon request. We strongly encourage code deposition in a community repository (e.g. GitHub). *Nature Methods* guidance for providing algorithms and software for publication provides further information on this topic.

Materials and reagents

Policy information about availability of materials

8. Materials availability

Indicate whether there are restrictions on availability of unique materials or if these materials are only available for distribution by a third party.	No unique materials were used.

9. Antibodies

Describe the antibodies used and how they were validated for use in the system under study (i.e. assay and species).	Antibodies used in this study are listed in the supplementary information. The antibodies were characterized by manufactures.

10. Eukaryotic cell lines

a. State the source of each eukaryotic cell line used.	HeLa, A549 cells were provided by Prof. T. Carell and Prof. A. Tietze respectively; SH-SY5Y (CRL-226) purchased from Sigma. Human iPSCs were prepared by reprogramming newborn foreskin fibroblasts CRL2522 (ATCC). As feeders NuFF3-RQ IRR human newborn foreskin fibroblasts (GSC-3404, GlobalStem) were used.
b. Describe the method of cell line authentication used.	IPSCs pluripotency was confirmed and the culture was manually cleaned from any differentiated cells whenever necessary.
c. Report whether the cell lines were tested for mycoplasma contamination.	HeLa, SH-SY5Y, A549 cells were tested for mycoplasma contamination and are negative.<br>iPSCs are regularly tested for mycoplasma contamination and are negative.<br>NPCs and neurons were derived from the repogrammed, mycoplasma-negative iPSCs.
d. If any of the cell lines used are listed in the database of commonly misidentified cell lines maintained by ICLAC, provide a scientific rationale for their use.	No commonly misidentified cell lines were used in the study.

Animals and human research participants

Policy information about studies involving animals; when reporting animal research, follow the ARRIVE guidelines

11. Description of research animals

Provide all relevant details on animals and/or animal-derived materials used in the study.	No animals were used.

Policy information about studies involving human research participants

12. Description of human research participants

Describe the covariate-relevant population characteristics of the human research participants.	The study did not involve the human research participants.
