# Supplementary material for: FICD activity and AMPylation remodelling modulate human neurogenesis
Source: Nat Commun. 2020 Jan 24;11:517. doi: 10.1038/s41467-019-14235-6 (PMC6981130; doi:10.1038/s41467-019-14235-6)
Supplement: Supplementary file 3 — Description of Additional Supplementary Files [file 41467_2019_14235_MOESM3_ESM.pdf]

Description of Additional Supplementary Files

File name: Supplementary Data 1
Description: Pro-N6pA significantly enriched proteins; Summary of significantly enriched proteins in studied cell types.

File name: Supplementary Data 2
Description: Olaparib treatment; Table of enriched proteins from HeLa cells treated with Olaparib and pro-N6pA probe.

File name: Supplementary Data 3
Description: ADP-ribosylation reference list; Reference list of the known ADP-ribosylated proteins.

File name: Supplementary Data 4
Description: AMPylation sites; Table of all identified AMPylation sites.

File name: Supplementary Data 5
Description: Previously identified AMPylated proteins; Reference list of previously identified AMPylated proteins.

File name: Supplementary Data 6
Description: FICD OX and KD_HeLa; Table of enriched proteins from HeLa cells overexpressing FICD and FICD knockdown HeLa cells.

File name: Supplementary Data 7
Description: FICD interacting proteins; Table of enriched FICD interacting proteins by using DSSO crosslinking reagent.

File name: Supplementary Data 8
Description: Chemical-proteomics complete result files; Table containing complete lists of MaxQuant identified proteins from chemical-proteomic experiments.

File name: Supplementary Data 9
Description: Cellular localization of AMPylated proteins; Table describing subcellular localization of enriched proteins.

File name: Supplementary Data 10
Description: FICD OX and KD_SH-SY5Y whole proteome; Table summarizing the whole proteome analysis from SH-SY5Y cells transiently overexpressing FICD and FICD knockdown SH-SY5Y cells.

File name: Supplementary Data 11
Description: FICD OX_SH-SY5Y enrichment; Table of enriched proteins from SH-SY5Y cells transiently overexpressing FICD and FICD knockdown SH-SY5Y cells.

File name: Supplementary Data 12
Description: GO Terms; Complete lists of GO term analyses.
